# Vascularization and Engraftment of Transplanted Human Cerebral Organoids in Mouse Cortex

**DOI:** 10.1523/ENEURO.0219-18.2018

**Published:** 2018-11-20

**Authors:** Nicolas Daviaud, Roland H. Friedel, Hongyan Zou

**Affiliations:** 1 Fishberg Department of Neuroscience and Friedman Brain Institute, New York, NY 10029,; 2 Department of Neurosurgery, Icahn School of Medicine at Mount Sinai, New York, NY 10029

**Keywords:** cerebral organoid, CNS injury, neural stem cell transplant, vascularization of intracerebral graft

## Abstract

Neural stem cells (NSCs) hold great promise for neural repair in cases of CNS injury and neurodegeneration; however, conventional cell-based transplant methods face the challenges of poor survival and inadequate neuronal differentiation. Here, we report an alternative, tissue-based transplantation strategy whereby cerebral organoids derived from human pluripotent stem cells (PSCs) were grafted into lesioned mouse cortex. Cerebral organoid transplants exhibited enhanced survival and robust vascularization from host brain as compared to transplants of dissociated neural progenitor cells (NPCs). Engrafted cerebral organoids harbored a large NSC pool and displayed multilineage neurodifferentiation at two and four weeks after grafting. Cerebral organoids therefore represent a promising alternative source to NSCs or fetal tissues for transplantation, as they contain a large set of neuroprogenitors and differentiated neurons in a structured organization. Engrafted cerebral organoids may also offer a unique experimental paradigm for modeling human neurodevelopment and CNS diseases in the context of vascularized cortical tissue.

## Visual Abstract

**Figure:**
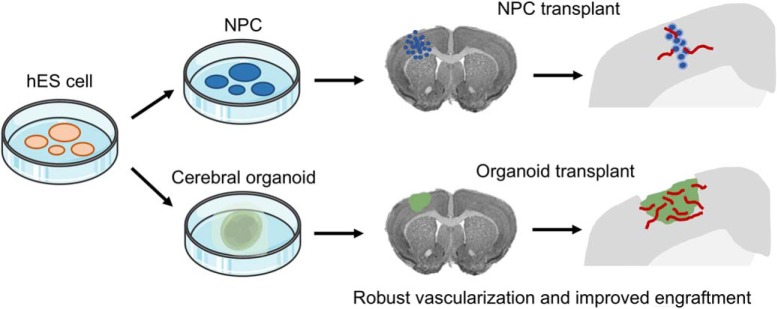

## Significance Statement

Neural stem cells (NSCs) hold great promise for neural repair, but conventional cell-based transplant methods face the hurdles of poor graft survival and inadequate neural differentiation. Here, we transplanted cerebral organoids derived from human pluripotent cells into lesioned mouse cortex. We report enhanced survival, robust vascularization, and multilineage differentiation of engrafted human cerebral organoids in host mouse brain. Cerebral organoid transplantation therefore represents an alternative, tissue-based transplantation strategy for neural repair and for modeling human neurodevelopment and CNS diseases in the context of vascularized cortical tissue.

## Introduction

CNS injury or degeneration results in devastating neurologic deficits from irreversible loss of neurons. Functional compensation from surviving neural networks and reparative efforts from endogenous neural stem cells (NSCs) are limited in their efficacy. Cell replacement therapy has thus been explored for reconstruction of neural circuits since the early 1970s (Das and Altman, 1972), and the interest has been reignited with the advent of induced pluripotent stem cells (iPSCs; Takahashi and Yamanaka, 2006) and improved neuronal differentiation protocols (Thompson and Björklund, 2015; Grade and Götz, 2017). Grafted NSC can undergo neuronal and glial differentiation, and display neurite outgrowth (Snyder et al., 1997). However, engraftment rate, long-term survival, and neuronal differentiation remain limited. Hence, novel strategies are needed to advance cell replacement therapy as a viable treatment option for CNS injury or degeneration.

Contrary to the poor engraftment rate of implanted dissociated neural progenitor cells (NPCs), mouse embryonic cortical tissue transplants survive well and show successful integration in adult mouse brain with establishment of substantial connectivity with host targets (Gaillard et al., 2007). The proof-of-principle that neural tissue replacement can work in human has been provided by the success of intrastriatal transplantation of human fetal mesencephalic tissue for Parkinson’s disease (PD) patients (Kordower et al., 1995). Clinical trials have since been conducted for fetal tissue transplant in PD patients, demonstrating long-term safety and clinical benefits (Lazic and Barker, 2003; Barker et al., 2013). Therefore, a shift from cell-based to tissue-based transplantation represents a promising strategy, but the scarcity of fetal tissues and ethical concerns limit the development of such an approach (Lindvall et al., 2004).

Recently, a novel three dimensional (3D) culture method has been developed to differentiate human PSCs into cerebral organoids (Lancaster et al., 2013; Lancaster and Knoblich, 2014). Cerebral organoid culture taps into the enormous self-organizing capacity of embryonic stem cells (ESCs)/iPSCs to form complex tissue structures under defined feeder cell-free conditions, with no addition of exogenous patterning cues or morphogens, an important safety point for transplantation. After 30 d of culture in Matrigel droplets under rotary condition, cerebral organoids adopt a predominantly dorsal forebrain regional specification, containing fluid-filled ventricle-like structures that are aligned with Sox2+ neuroprogenitors in a ventricular/subventricular-like zone (VZ/SVZ) and doublecortin (DCX)+ neuroblasts in an outer layer. Rudimentary cortical stratification takes place after longer culture periods, with cortical neurons differentiating into pyramidal identities displaying glutamatergic receptor activity and efferent long-range axons in a stereotypical inside-out stratified layout (Lancaster et al., 2013; Lancaster and Knoblich, 2014). Cerebral organoids thus provide a new experimental platform to study human brain development and to model CNS disorders such as microcephaly (Lancaster et al., 2013; Li et al., 2017), autism spectrum disorders (Forsberg et al., 2018), and Zika virus infection (Qian et al., 2016; Watanabe et al., 2017).

We hypothesized that transplantation of cerebral organoids derived from human ESC (hESC)/iPSC may enhance graft survival, neural differentiation, and integration in host brain as compared to conventional cell-based transplantation for the following reasons. First, the 3D cellular arrangement of the cortical plate-like tissue in cerebral organoids may provide a protective shield against hostile elements at the graft site. Second, a neuroprecursor pool in the cerebral organoids residing in protected stem cell niches in the VZ/SVZ may serve as a source for neurogenesis and stem cell-derived trophic factors. Third, a rudimentary 3D cortical structure is already in place at the time of transplant, enabling intrinsic patterning cues to direct organized neuronal differentiation and prevent aberrant proliferation and lineage progression.

Here, we performed comprehensive side-by-side comparisons of transplantation of dissociated NPC versus cerebral organoids, both derived from identical hESC cultures. Postnatal day (P)8–P10 mice were used as recipients and frontoparietal cortex as transplant site. We compared graft survival, vascularization, NSC population, and neurodifferentiation at two and four weeks after transplantation. We found enhanced survival of cerebral organoid transplants as compared to grafted NPC, and robust vascularization of the organoid grafts from host vessels. There were also abundant neuroprogenitors and evidence for multilineage differentiation in the engrafted cerebral organoids. A recent study by Mansour et al. (2018) also tested intracerebral grafting of hPSC-derived brain organoids. In that study, adult nonobese diabetic-severe combined immunodeficient (NOD-SCID) mice were used as recipients and retrosplenial cortex was selected as transplant site based on the rich vascularized surface overlaying this area. They reported that organoid grafts showed successful engraftment with robust vascularization from host brain, and in long-term analysis of up to eight months after transplant, progressive neuronal differentiation and maturation, long-range axon projections, and functional graft-to-host synaptic connectivity were observed. Our data thus echo those of Mansour et al. (2018) in demonstrating the practicality of transplantation of hiPSC-derived cerebral organoids as a promising alternative for cell replacement therapy for CNS injury and neurodegeneration. Transplantation of cerebral organoids also provides a unique experimental paradigm to study human neurodevelopment and to model CNS diseases in the context of vascularized cortical tissue.

## Materials and Methods

### Animal care

P8–P10 CD1 mice of either sex (Charles River Laboratories) were used as transplant recipients for analysis up to four weeks after grafting without immunosuppressive treatment, as immunosuppression was only mandatory to achieve engraftment beyond two months (Espuny-Camacho et al., 2013). Mice were group-housed and kept in a 12/12 h light/dark cycle with free access to food and water *ad libitum*. All animal procedures were performed in accordance with the IACUC committee of Icahn School of Medicine at Mount Sinai animal care committee’s regulations.

### hESC culture

Human pluripotent ESCs were provided by WiCell (H9 hES cells, WAe009-A). For hESC culture, six-well plates were coated with diluted Matrigel (growth factor reduced; 1:100, BD Biosciences) for 20 min at 37°C, and cells were plated and cultured in mTeSR1 media (STEMCELL Technologies) supplemented with 2 µM ROCK inhibitor Thiazovivin (Millipore) for 24 h. Cells were cultured with media changed every day until ready to passage or harvest in mTESR1 media (without ROCK inhibitor).

### GFP labeling of hESCs

H9 hES cells were infected with lentivirus EF1a-GFP-IRES-Puro, followed by puromycin selection (1 µg/ml, Thermo Fisher Scientific). For lentivirus preparation, the pEGIP lentivirus plasmid (Addgene plasmid #26777) was transfected into 293T cells together with envelope plasmid pMD2.G and packaging plasmid psPAX2 (Addgene #12259 and #12260) with X-tremeGENE 9 DNA transfection reagent (Roche). Lentiviruses were concentrated from culture media supernatant 72 h after transfection by ultracentrifugation.

### Generation of hNPC from hESCs

hES cells were cultured in low attachment 96-well plates for 4 d to generate embryoid bodies (EBs). EBs were then transferred to Matrigel-coated six-well plates for attachment and further cultured in neural induction medium (STEMdiff, STEMCELL Technologies) for 4–5 d. Next, cells were plated on laminin-coated (10 µg/ml, Thermo Fisher Scientific) six-well plates and cultured in human NSC medium (NeuroCult, STEMCELL Technologies) supplemented with 20 ng/ml of epidermal growth factor (EGF) and 10 ng/ml of basic fibroblast growth factor (bFGF; Peprotech) for 7 d for NPC maturation, which were then used for transplant.

For proliferation and differentiation assays, 12-mm glass coverslips were pre-coated with 50 µg/ml poly-D-lysine (PDL; Sigma Aldrich) and 10 µg/ml laminin (Thermo Fisher Scientific). NPC were seeded on coverslips at 12 000 cells/cm^2^ density and cultured in human NSC medium (NeuroCult, STEMCELL Technologies) supplemented with 20 ng/ml of EGF and 10 ng/ml of bFGF (Peprotech). For differentiation assays, cells were cultured in the same media, but with withdrawal of mitogens (EGF, bFGF) for 5 d. Cells were ﬁxed with 4% paraformaldehyde (pH 7.4, Acros Organics) in PBS at 4°C for 15 min and analyzed by immunoﬂuorescence.

### Cerebral organoid generation from hESCs

Human cerebral organoids were generated as described (Lancaster et al., 2013; Lancaster and Knoblich, 2014), with modifications as follows: human ES cells were detached using 50 µM EDTA (Thermo Fisher Scientific) and plated in round bottom ultra-low attachment 96-wells plate (CLS7007, Corning) at a density of 9000 cells per well in mTESR1 media (STEMCELL Technologies) supplemented with 1% antibiotics (penicillin streptomycin, Thermo Fisher Scientific) for a total of 6 d. During the first 4 d of the culture, media were supplemented with 10 µM Thiazovivin. Half of the media was changed every day. After 6 d of culture or when embryonic bodies (EBs) reached ∼500–600 μm in diameter and when surface tissue began to brighten and formed smooth edges, media were switched to a neural induction media (Stemdiff, STEMCELL Technologies). Half of the media was changed every day for 3–4 d. After neuroepithelium emerged (usually at approximately day 9–10), organoids were embedded in Matrigel droplets (25 µl, BD Biosciences) and cultured in 6 cm Petri dishes (Falcon) for 4 d in cerebral organoid differentiation media consisting of 1:1 DMEM-F12 and Neurobasal media (Gibco), with addition of 0.5% N2 supplement (Life Technologies), 0.5% ml MEM-NEAA (Gibco), 1% Glutamax (Gibco), 1% B27 supplement without vitamin A (Life Technologies), 0.1 µM of 2-mercaptoethanol (Millipore), 2.6 µg/ml insulin (Sigma Aldrich), and 1% pen/strep antibiotics (Gibco). After 4 d, the organoid Matrigel droplets were cultured with addition of vitamin A on an orbital shaker (VWR) at 85 rpm for four additional weeks, and then used for transplant.

### Grafting of NPC or cerebral organoid into mouse cortex

Before transplantation, a quality control of cerebral organoids was performed by brightfield microscopy to select the organoids that displayed an appropriate differentiation/maturation phenotype without massive cyst formation or premature differentiation. Then, a measurement of organoid size was performed by brightfield microscopy and organoids of similar sizes were selected for transplant. To estimate the cell numbers in organoids at time of transplant, organoids were dissociated by 15-min trypsin incubation, and cells were counted by Trypan blue exclusion.

P8–P10 mice were anesthetized with isoflurane and secured on a stereotactic frame (Stoelting). Scalp was opened on the left hemisphere. Using a restricted depth stab knife, an ∼1 × 1 mm craniotomy window was opened, with the bone flap hinged on anterior base. A cortical lesion was made by removing ∼1 mm^3^ piece of the frontoparietal cortex. Using a pair of forceps, one cerebral organoid at 42 d *in vitro* culture was implanted into the lesioned mouse cortex. The craniotomy window was closed by returning the bone flap to the original position and sealed with fibrin glue (Evicel, Fibrin Sealant), followed by skin closure with sutures.

### Intracerebral implantation of NPC

NPC differentiated from H9 hES cells were dissociated with trypsin, counted, and assessed for viability by Trypan blue exclusion. Cells were washed twice with DMEM, and then suspended in DMEM at a concentration of 5 × 10^4^/μl. Using a 5-μl Hamilton syringe (Hamilton glass syringe 600 series RN) and a 26-gauge needle, 1 × 10^5^ NPC (2 μl) were implanted into the left frontoparietal hemisphere. To be consistent with the organoid transplant location, the coordinates used for the intracerebral injection were 1 mm posterior to the bregma, 1 mm lateral to the sagittal suture (left hemisphere), and 1 mm below the dura.

### Histologic analyses

Two or four weeks after transplantation, animals received a lethal dose of pentobarbital (390 mg/ml) and transcardial perfusion was performed with 50-ml cold PBS, followed by 50-ml cold 4% PFA/PBS. Brains were removed and post-fixed overnight in 4% PFA/PBS and cryoprotected for 48 h in 30% sucrose. Brains were then snapped freeze in isopentane (Sigma Aldrich) and embedded in O.C.T. compound (Tissue-plus, Fisher Healthcare) and stored at –80°C. Brain tissues were sectioned into 14-µm coronal sections with cryostat (CM1850, Leica). For immunofluorescence analysis, non-specific binding sites were blocked with 4% BSA in PBS (Fisher Bioreagents), 0.2% Tween (Tween 20, Acros Organics), and 10% normal donkey serum (Jackson ImmunoResearch) for 1 h at room temperature, and slices were then incubated with the following antibodies diluted in 4% BSA/PBS, 0.2% Tween: rabbit anti-activated caspase 3 (AC3; Abcam, ab2302, 1:100); rat anti-mouse CD31 (BD Biosciences, 553370, 1:200); rat anti-mouse CD45 (BD Biosciences, 550539, 1:200); guinea pig anti-DCX (Millipore AB2253, 1:500); mouse anti-GalC (Millipore MAB342, 1:200); rabbit anti-GFAP (Invitrogen 180063, 1:200); chicken anti-GFP (Aves Lab, GFP-1020, 1:300); rabbit anti-Iba1 (Wako Chemicals, 019-19741, 1:200); rabbit anti-Ki67 (Abcam, ab15580, 1:500); mouse anti-MTCO_2_ (Mitochondrially Encoded Cytochrome C Oxidase II; Abcam, ab110258, 1:100); rabbit anti-Nanog (Abcam, ab109250, 1:300); chicken anti-Neurofilament H (NF-H, Abcam Ab5539, 1:300); mouse anti-Oct4 (Abcam, ab184665, 1:300); rabbit anti-Olig2 (Millipore AB9610, 1:500); rabbit anti-SOX2 (Millipore AB5603, 1:200), rabbit anti-TBR1 (Abcam ab31940, 1:500); rabbit anti-TBR2 (Abcam ab23345, 1:500), and mouse anti-tubulin β-III (Tuj1, R&D Systems MAB1195, 1:100). Slides were then washed in PBS with 0.1% Tween and detection was performed with Alexa Fluor-coupled secondary antibodies (Invitrogen and Jackson ImmunoResearch) and DAPI nuclear counterstain (Invitrogen).

Quantifications were performed on at least two brain slices per animal and three independent mice for each condition. The two slices were separated by ∼150 µm. We selected the sections that were in the middle of the transplant area. Grafts were outlined by GFP or hMito (human mitochondria marker) immunofluorescence. For each selected 14-µm graft-bearing brain slice, we quantified different markers within the entire GFP or hMito-labeled area by Fiji software (Schindelin et al., 2012) unless otherwise specified.

### Statistical analysis

For each experiment, the number of mice used in each cohort, and the number of images analyzed from each animal are listed in figure legends. Data are presented as mean value with SEM, unless otherwise stated. At least three independent grafts in three different animals were tested and at least two representative images from each animal were quantified.

Cluster-based summary statistics using within-subject averaging were performed whenever possible. GraphPad Prism 7 was used for statistical analyses. Differences between conditions were determined using a two-way ANOVA test, followed by a Tukey *post hoc* test. For individual comparison within the group, normality of data was assessed using a Shapiro–Wilk test followed by a Student’s *t* test. Results were considered significant for *p* < 0.05 (Table 1).

**Table 1.** Statistical analyses

	Data structure	Type of test	Condition	Power
Fig. 3*B*	Dataset with more than two groups	Two-way ANOVA - Tukey *post hoc* test	Two weeks:organoids vs two weeks:NPCs	*p = 0.0393
			Four weeks:organoids vs four weeks:NPCs	n.s., *p* = 0.7729
Fig. 3*D*	Dataset with more than two groups	Two-way ANOVA - Tukey *post hoc* test	Two weeks:organoids vs two weeks:NPCs	n.s., *p* = 0.1836
			Four weeks:organoids vs four weeks:NPCs	n.s., *p* = 0.9634
Fig. 5*B*, left	Dataset with more than two groups	Two-way ANOVA - Tukey *post hoc* test	Two weeks:organoids vs two weeks:NPCs	n.s., *p* = 0.1059
			Four weeks:organoids vs four weeks:NPCs	*p = 0.0293
			Four weeks:organoids vs two weeks:organoids	n.s., *p* = 0.4072
Fig. 5*B*, right	Dataset with more than two groups	Two-way ANOVA - Tukey *post hoc* test	Two weeks:organoids vs two weeks:NPCs	n.s., *p* = 0.6113
			Four weeks:organoids vs four weeks:NPCs	n.s., *p* = 0.7485
			Four weeks:organoids vs two weeks:organoids	n.s., *p* = 0.2955
Fig. 6*B*, top	Dataset with more than two groups	Two-way ANOVA - Tukey *post hoc* test	Two weeks:organoids vs two weeks:NPCs	**p = 0.0014
			Four weeks:organoids vs four weeks:NPCs	*p = 0.0108
			Four weeks:organoids vs two weeks:organoids	n.s., *p* = 0.9757
Fig. 6*B*, bottom	Dataset with more than two groups	Two-way ANOVA - Tukey *post hoc* test	Two weeks:NPC vs two weeks:Organoid	*p = 0.0385
			Four weeks:NPC vs four weeks:Organoid	n.s., *p* = 0.2141
			Four weeks:organoids vs two weeks:organoids	n.s., *p* = 0.998
Fig. 6*D*, top	Dataset with more than two groups	Two-way ANOVA - Tukey *post hoc* test	Two weeks:organoids vs two weeks:NPCs	n.s., *p* = 0.1664
			Four weeks:organoids vs four weeks:NPCs	n.s., *p* = 0.7125
			Four weeks:organoids vs two weeks:organoids	n.s., *p* = 0.3625
Fig. 6*D*, bottom	Dataset with more than two groups	Two-way ANOVA - Tukey *post hoc* test	Two weeks:organoids vs two weeks:NPCs	n.s., *p* = 0.9555
			Four weeks:organoids vs four weeks:NPCs	n.s., *p* = 0.9037
			Four weeks:organoids vs two weeks:organoids	n.s., *p* = 0.2857
Fig. 8*B*	Dataset with more than two groups	Two-way ANOVA - Tukey *post hoc* test	Two weeks:organoids vs two weeks:NPCs	n.s., *p* = 0.2590
			Four weeks:organoids vs four weeks:NPCs	*p = 0.0311
			Four weeks:organoids vs two weeks:organoids	n.s., *p* = 0.1724
Fig. 8*D*	Dataset with more than two groups	Two-way ANOVA - Tukey *post hoc* test	Two weeks:organoids vs two weeks:NPCs	n.s., *p* = 0.6897
			Four weeks:organoids vs four weeks:NPCs	***p = 0.0006
			Four weeks:organoids vs two weeks:organoids	**p = 0.0041
Fig. 8*F*	Dataset with two groups	Shapiro–Wilk followed by a Student’s *t* test	Two weeks:organoids vs four weeks:organoids	n.s., *p* = 0.3437

## Results

### hESC-derived NPC and cerebral organoids for intracerebral transplantation

**Figure 1.**
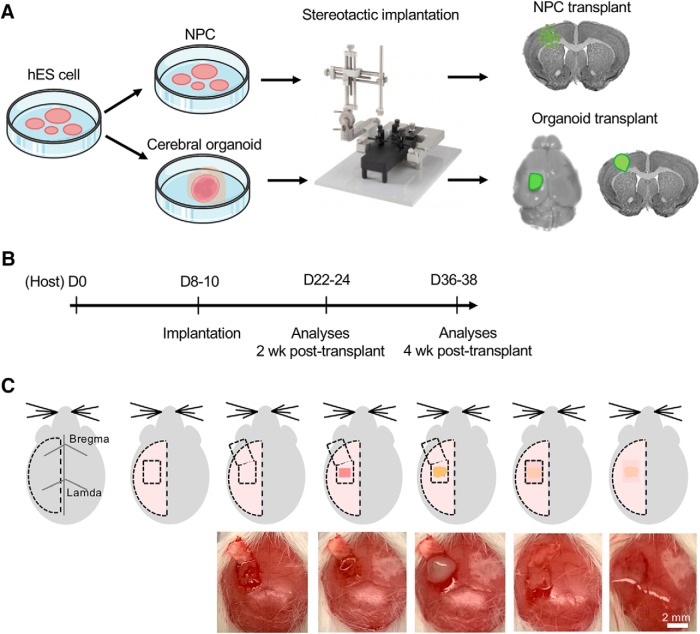
Schematic depiction of intracranial transplantation of human cerebral organoids. ***A***, hES cells were differentiated into NPC or cerebral organoids. Stereotactic surgery was performed to transplant one single cerebral organoid in the lesioned frontoparietal cortex in P8–P10 mice. For NPC transplantation, dissociated NPC were implanted into identical cortical region by stereotactic injection. ***B***, Experimental timeline. P8–P10 immunocompetent mice were used as recipients for transplantation of dissociated human NPC or human cerebral organoids. Histologic analyses of the grafts were performed two or four weeks after grafting. ***C***, Schematic diagrams (top) and intraoperative photographs (bottom) of cerebral organoid transplantation. Briefly, from left to right, once scalp is reflected, a small craniotomy window was opened with the bone flap hinged at the anterior base, and a small piece of the frontoparietal cortex (∼1 mm^3^) was removed. A single cerebral organoid was then implanted into the prelesioned mouse cortex, followed by return of the bone flap to close the craniotomy window, sealed with fibrin glue, followed by scalp closure.

For side-by-side comparisons, we differentiated the same batch of hESC into either NPC or cerebral organoids and performed stereotactic transplantation into left frontoparietal cortex of immunocompetent P8–P10 mice (Fig. 1*A*). We compared graft survival, vascularization, NSC pool, and neurodifferentiation at two and four weeks after transplantation (Fig. 1*B*). To ensure that the organoid grafts remained inside the lesion cavity following transplantation, we optimized the technique of opening a small craniotomy window with a bone flap hinged at the anterior base, and after transplantation, the bone flap was returned to original position, sealed with fibrin glue, followed by scalp closure (Fig. 1*C*).

**Figure 2.**
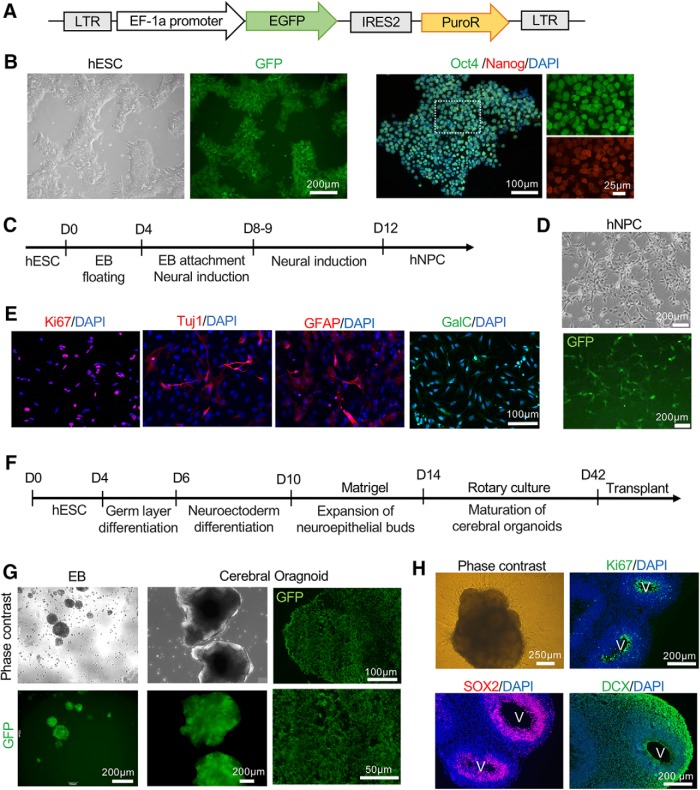
Characterization of GFP-labeled NPC and cerebral organoids derived from hESC. ***A***, Diagram of lentiviral vector expressing EGFP (enhanced GFP) driven by EF-1a promoter to label hES cells. ***B***, Phase-contrast and fluorescent images of GFP-labeled hES cells (left) and expression of pluripotency markers Oct4 (green) and Nanog (red; right). ***C***, Timeline of differentiation of hES cells into NPC. ***D***, Phase-contrast and fluorescent images of human NPC derived from GFP-labeled hES cells. ***E***, Representative immunofluorescence images of NPC stained for proliferation marker Ki67 and the indicated neural markers. ***F***, Timeline of derivation of cerebral organoids from hESC. ***G***, left panels, Phase-contrast and fluorescence images of GFP-labeled EB or Matrigel-embedded cerebral organoids. Right panel, Immunofluorescent images of sectioned cerebral organoids stained for GFP. ***H***, Phase-contrast image of cerebral organoid at day 42 of culture and immunofluorescent images of sectioned day 42 cerebral organoid stained for the indicated markers. V: ventricle-like structures. Note the proliferative zone (Ki67+) and Sox2+ neuroprogenitors at the VZ/SVZ, and DCX+ neuroblasts in the outer layer.

To facilitate visualization of donor cells in host cortical tissue, we generated a hESC line expressing green fluorescent protein (GFP) ubiquitously (Zou et al., 2009; Fig. 2*A*). After lentiviral infection and puromycin selection, GFP-expression of hESC was assessed, while their pluripotency was verified by immunocytochemistry with uniform expression of stemness markers Oct4 and Nanog (Fig. 2*B*). GFP-positive hESC were then differentiated into hNPC by way of EB formation followed by neural induction (Fig. 2*C*,*D*). To confirm multipotency of hNPC, differentiation condition was applied for 5 d by mitogen withdrawal, and immunocytochemistry revealed expression of TUJ1 (neuronal marker), GFAP (astrocyte marker), and GalC (oligodendrocyte marker) while most cells remained proliferative as shown by the proliferation marker Ki67 (Fig. 2*E*). In parallel, the same batch of GFP-expressing hESC were used to generate cerebral organoids (Lancaster et al., 2013; Lancaster and Knoblich, 2014). GFP expression was confirmed at each stage of organoid development (Fig. 2*F*,*G*). After 42 d of *in vitro* culture, with the last 28 d under rotary condition embedded in Matrigel droplet, cerebral organoids adopted a predominantly dorsal forebrain specification, containing multiple ventricle-like structures aligned with SOX2+ neuroprogenitors in the VZ/SVZ zone and DCX+ neuroblasts in the outer layer (Fig. 2*H*).

### Enhanced survival of cerebral organoid transplants

We grafted one single hESC-derived cerebral organoid into the lesion cavity in the left frontoparietal cortex of P8–P10 CD1 mice. We determined that the transplanted organoids were composed of an average of 2.5 × 10^5^ cells (±1.4 × 10^5^ SEM), with around 22% of cells being Sox2+ neuroprogenitors residing in VZ/SVZ-like structures and the rest of the cells in various stages of neurodifferentiation. In parallel, 1 × 10^5^ dissociated hNPC were implanted into identical cortical location by stereotactic injection, so that comparable number of neuroprogenitors were transplanted in both graft types. At two and four weeks after transplantation, all recipient mice survived the procedure and the transplanted organoids or NPC could be found in each host mouse.

**Figure 3.**
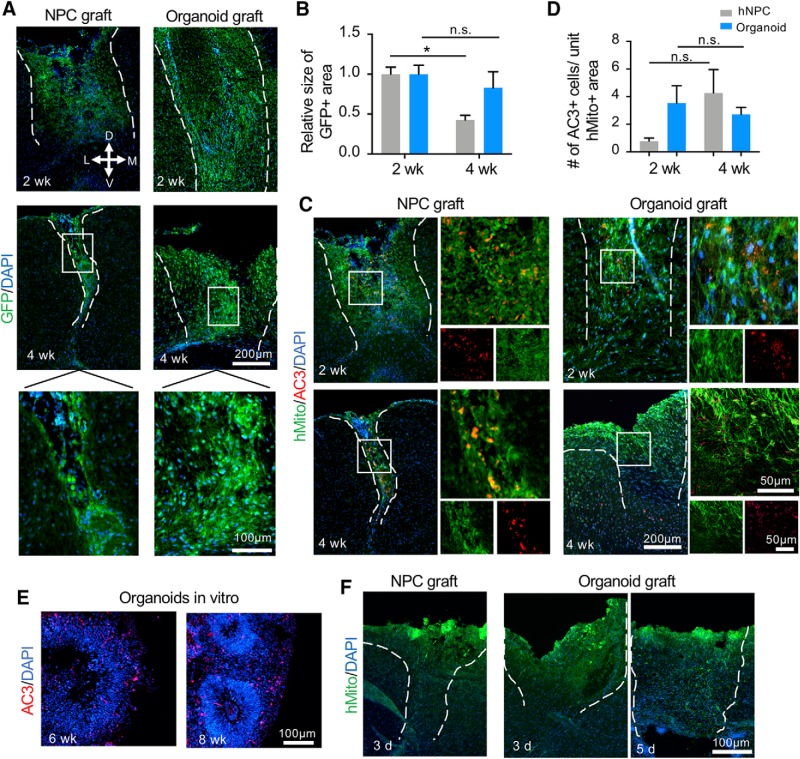
Engraftment and survival of NPC and cerebral organoid transplants. ***A***, Representative immunofluorescence images of NPC transplant (left) and cerebral organoid transplant (right) at the indicated time points post-grafting. Enlarged images of the boxed area are shown at the bottom. D: dorsal, V: ventral, M: medial, L: lateral. ***B***, Quantification showing the relative size of GFP-positive area of NPC and organoid transplants at two and four weeks after grafting. A significant decrease of the graft size of the NPC transplants was detected at four weeks as compared to two weeks after grafting. ***C***, Representative immunofluorescence images for hMito (human mitochondria) and AC3 in NPC transplants (left) or in cerebral organoid transplants (right) at the indicated time points after transplantation. ***D***, Quantification showing the number of AC3-positive apoptotic cells per unit area of hMito-positive grafts at the indicated time points. ***E***, Representative immunofluorescence images showing a high number of apoptotic cells (AC3+) in stage-matched cerebral organoids in culture at six and eight weeks. ***F***, Representative immunofluorescence images of NPC transplant (left) and cerebral organoid grafts (right) 3 or 5 d after transplantation. At these early time points, grafts had not yet been firmly integrated into host brains. Dashed white lines delineate the graft areas; *p < 0.05; n.s., non-statistically significant. Two-way ANOVA followed by a Tukey *post hoc* test; *n* = 3 mice for each time point and two images from each mouse.

To compare engraftment rate, we first measured the size of the graft areas as demarcated by GFP-labeled grafted cells. Consistent with earlier reports of poor survival of transplanted NSC in dissociated state (Johann et al., 2007), NPC grafts displayed significant shrinkage from two- to four-week time periods after transplant (*p* = 0.039), whereas the size of cerebral organoid grafts remained stable between two and four weeks after transplantation (Fig. 3*A*,*B*). In some instances, organoids grafts appeared fragmented, likely a result of technical difficulty (transplanting a relatively large human cerebral organoid into a small host mouse brain) or cell death from trauma, hypoxia, and inflammation before engraftment took place. Taken together, our finding of an enhanced survival of organoid grafts supports the hypothesis that structured cellular arrangement in tissue-based transplants provides better protection of donor cells from the hostile graft microenvironment.

We next examined the extent of apoptosis in NPC versus organoid transplants by immunohistochemistry for AC3. We found no significant differences in the average number of AC3+ cells per unit GFP+ graft area between the two graft types at two weeks (*p* = 0.18) or four weeks (*p* = 0.96) after transplantation, although there were high variabilities (Fig. 3*C*,*D*). Notably, similar to the finding by Mansour et al. (2018), the number of apoptotic cells were much lower in organoid grafts than in stage-matched cultured organoids (Fig. 3*E*), which may reflect phagocytic clearing *in vivo*.

To better understand the time course of organoid engraftment, we performed additional short-term post-grafting analyses. We found that by 3 or 5 d after transplantation, organoid grafts had not yet been firmly integrated into host brain tissue as only ∼50% of the grafts were found in host animals after tissue processing, indicating insufficient time for engraftment (Fig. 3*F*).

**Figure 4.**
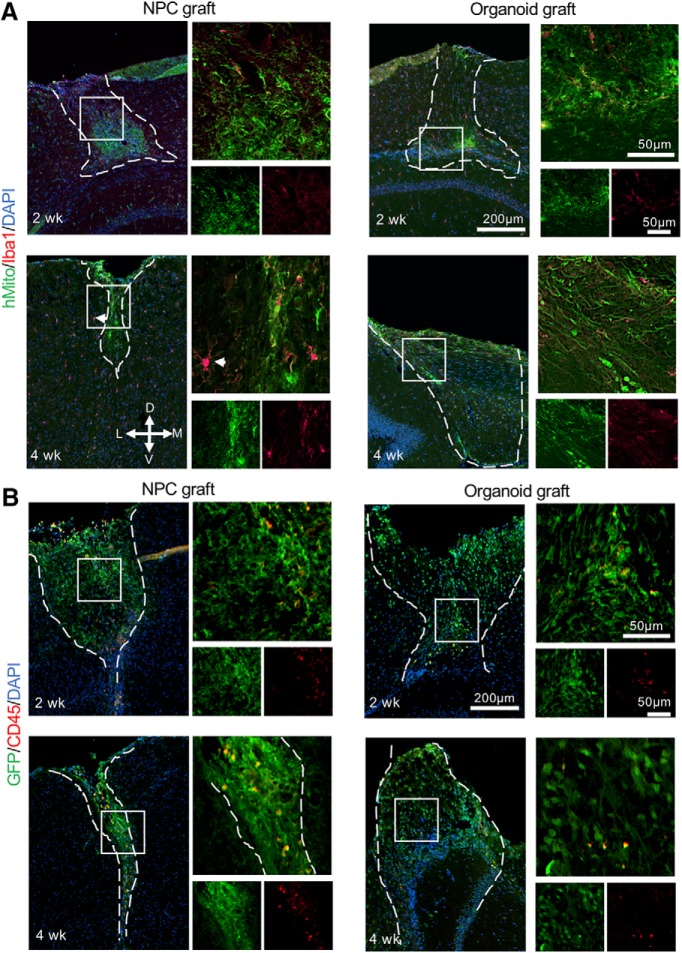
Host immune response following NPC and organoid transplants. ***A***, Representative immunofluorescence images of Iba1 in hMito-labeled grafts, at two and four weeks after transplantation. Note the hypertrophied Iba1+ microglia inside the NPC graft, as well as in host brain tissue adjacent to the NPC graft (white arrowhead). ***B***, Representative immunofluorescence images of CD45 in GFP-labeled grafts at indicated time points. Dashed white lines delineate the graft areas. Enlarged images of the boxed area are shown on the right. D: dorsal, V: ventral, M: medial, L: lateral.

We further examined host immune response against the grafts by immunohistochemistry for Iba1, a marker of activated microglia/macrophages. We detected low number of Iba1+ cells in both NPC and organoid transplants after two weeks; but after four weeks, the Iba1+ cells were increased and exhibited hypertrophied morphology in NPC transplants and adjacent host brain tissues, suggesting enhanced phagocytic activity. In contrast, Iba1+ cells remained relatively scant and small in size in organoid transplants after four weeks (Fig. 4*A*). The Iba1+ cells in the organoid grafts did not colocalize with hMito (human mitochondria marker), indicating host origin of the microglia within the grafts, similar to the data shown by Mansour et al. (2018). Similar results were observed using leukocyte common antigen CD45. At the transplant sites, we detected only scant CD45^hi^ cells in organoid grafts, and slightly more CD45^hi^ cells in NPC grafts, possibly indicating increased phagocytosis of dead donor cells within the NPC transplants (high regional variability precluded accurate quantification; Fig. 4*B*).

### Successful vascularization from host into the grafted cerebral organoids

**Figure 5.**
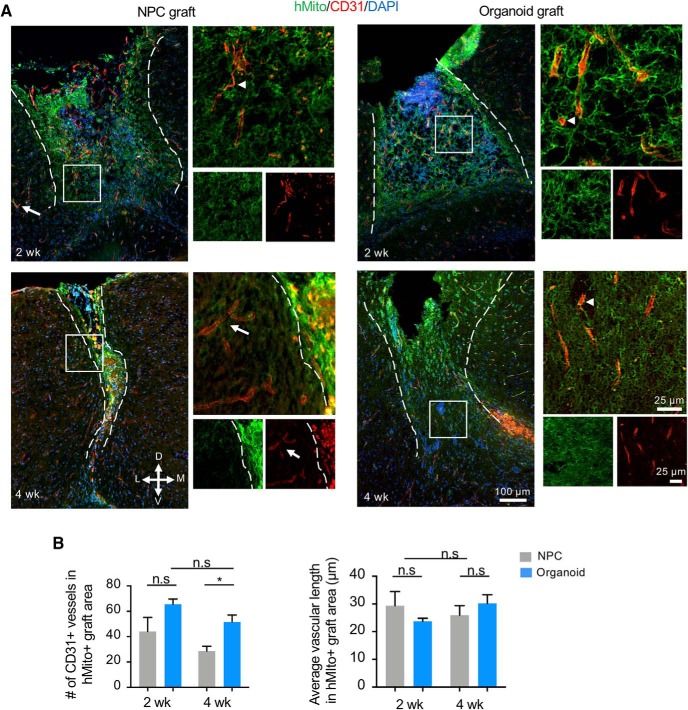
Vascularization of engrafted cerebral organoids. ***A***, Representative immunofluorescence images demonstrate penetration of host CD31-positive blood vessels into hMito+ NPC grafts (left panels) or cerebral organoid grafts (right panels) at the indicated time points post-transplantation. Notice that CD31-positive endothelial cells inside the grafts are hMito-negative (white arrowheads). White arrows: host vasculature. Dashed white lines delineate the graft areas. Enlarged images of the boxed area are shown on the right. D: dorsal, V: ventral, M: medial, L: lateral. ***B***, Quantifications of the number (left) and the average length (right) of CD31-positive blood vessels in hMito-labeled grafts demonstrate a higher number of vasculatures in the engrafted cerebral organoids compared to NPC transplants at four weeks after transplantation, but no significant difference in the average vascular length; *p < 0.05; n.s., non-statistically significant; *n* = 3 mice for each cohort, and at least two images analyzed from each mouse. Two-way ANOVA followed by a Tukey *post hoc* test.

Vascularization of the graft is a key determinant for successful integration into host tissue. We therefore compared the extent of graft vascularization by immunohistochemistry for CD31/PECAM1, an endothelial cell marker. We first quantified the number of blood vessels in the entire graft area as labeled by hMito immunostaining. We detected robust vascularization of the organoid transplants at both two and four weeks after grafting (Fig. 5*A*). Remarkably, CD31+ microvasculatures were present not only at the periphery, but also at the center of the grafts. Closer inspection revealed no apparent overlap between CD31 and hMito immunostaining, indicating host origin of the graft vasculature. From two to four weeks following transplantation, the number of microvasculatures in the engrafted organoids remained stable (Fig. 5*B*). In the NPC transplants, we also observed abundant CD31+ microvasculatures within the graft after two weeks; but after four weeks, a lower number of CD31+ blood vessels were detected (Fig. 5*A*). Quantification confirmed the presence of significantly more CD31+ vessels in organoid transplants than in NPC transplants at four weeks after grafting (*p* = 0.029). To verify that the higher number of blood vessels found in organoid transplants was not due to shorter or more branched vasculatures, we measured average vascular length, which was comparable between the two graft types (Fig. 5*B*). Notably, donor cells appeared disorganized in relation to vasculatures in NPC grafts, which may reflect their dissociated state at the time of transplant as compared to the tissue-like cellular arrangement in organoid transplants.

### Neuroprogenitor proliferation and neurodifferentiation in engrafted cerebral organoids

**Figure 6.**
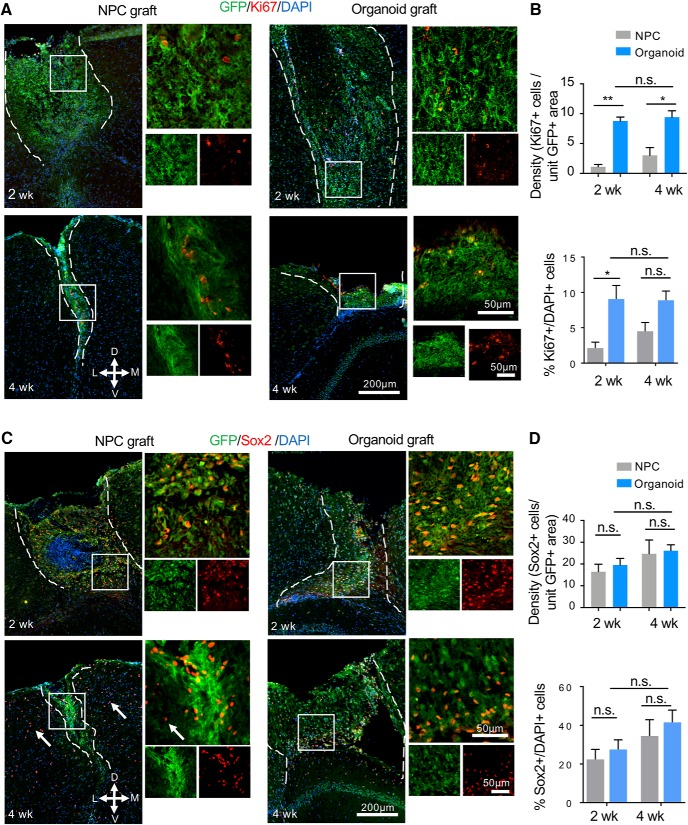
Cell proliferation and NSC pool in cerebral organoid transplants. ***A***, Representative immunofluorescence images of NPC (left panels) and cerebral organoid transplants (right panels) stained for GFP and proliferation marker Ki67. D: dorsal, V: ventral, M: medial, L: lateral. ***B***, Quantification (top) showing a higher density of Ki67+ cells per unit GFP+ area in cerebral organoid than in NPC transplants at both two and four weeks after transplantation. Bottom quantification: percentage of Ki67+/DAPI+ cells within the GFP+ cerebral organoid and NPC grafts. ***C***, Representative immunofluorescence images of NPC (left panels) and cerebral organoid transplants (right panels) showing abundant engrafted cells (GFP+) expressing stem cell marker Sox2 (red). White arrows: Sox2+ OPC in host cortical tissue. ***D***, Quantification (top) showing no significant difference of the density of Sox2+ cells per unit GFP+ area between NPC and cerebral organoid transplants at either time points. Bottom quantification: percentage of Sox2+/DAPI+ cells within the organoid and NPC grafts showing no significant difference between the two types of transplants at either timepoint. Dashed white lines delineate the graft areas. Enlarged images of the boxed area are shown on the right; *p < 0.05. **p < 0.01; n.s., non-statistically significant. Two-way ANOVA followed by a Tukey *post hoc* test; *n* = 3 mice for each time point and at least two images from each mouse.

**Figure 7.**
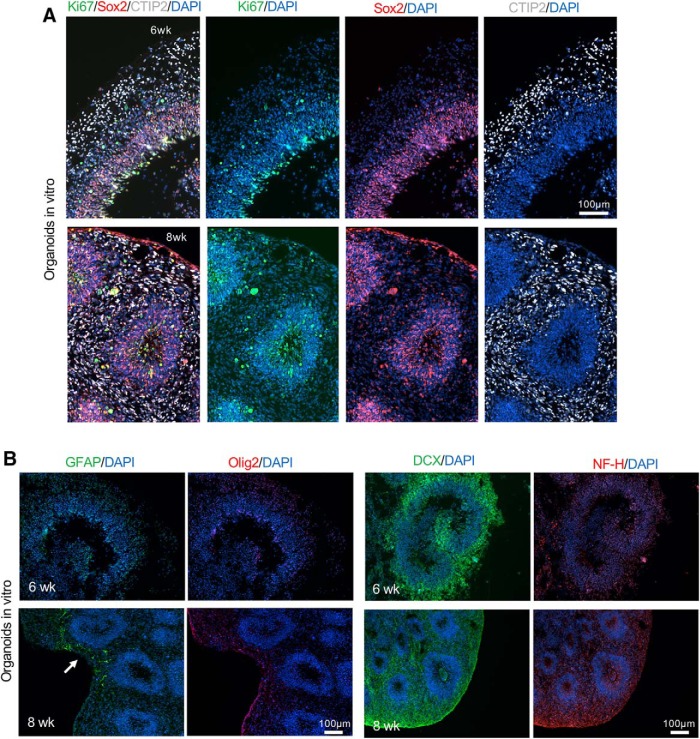
Stage-matched *in vitro* cerebral organoid characterization. ***A***, Representative immunofluorescence images of cultured cerebral organoids at six or eight weeks of maturation show layered organization of cortical-like tissue with proliferating cells (Ki67+) and NPC (SOX2+) mainly localized in the VZ/SVZ and neurons (CTIP2+) localized in the outer layer. ***B***, Representative immunofluorescence images of cerebral organoids after six or eight weeks of *in vitro* maturation. Left, A low number of astrocytes (GFAP+) was present in the organoids after eight weeks of maturation (white arrow), while no Olig2+ cells were detected. Right, Abundant neuroblasts (DCX+) were found at both time points while low level of expression of NF-H was detected at eight weeks of maturation.

We next examined proliferation of donor cells in the transplants using the proliferation marker Ki67. A significantly lower density of Ki67+ cells per unit GFP+ graft area was detected in NPC transplants as compared to organoid transplants at both two and four weeks after grafting (*p* = 0.0014 and *p* = 0.0108, respectively), while there was no significant difference in organoid transplants between the two time points (*p* = 0.97; Fig. 6*A*,*B*). The percentage of Ki67+/DAPI+ cells at two weeks after transplantation was also much lower in NPC transplants (∼2.5%) than in organoid transplants (∼9%; *p* = 0.038), but the difference at four weeks became more variable, thus not statistically significant (∼4.5% in NPC grafts and 9% in organoid grafts, *p* = 0.21; Fig. 6*B*). Of note, in NPC cultures, proliferative cells were abundant, even after 5 d under differentiation condition (shown in Fig. 2*E*); in contrast, in cultured organoids, Ki67+ cells were restricted to VZ/SVZ (Fig. 2*H*). Therefore, the starting number of proliferative cells in NPC transplants may in fact be higher than in organoids transplants. There was no significant difference in the percentage of Ki67+/DAPI+ cells in the organoid grafts between two and four weeks after grafting (*p* = 0.99). It is also notably that unlike in stage-matched cultured organoids where Ki67+ proliferative cells were detected predominantly in the progenitor zones aligning the ventricle-like structures (Fig. 7*A*), the Ki67+ cells in transplanted organoids appeared scattered throughout the grafts, reflecting disintegration of the ventricular structures following transplantation.

We also assessed neuroprogenitor populations in the two graft types. The Sox2+ neuroprogenitors remained abundant in both graft types, with comparable density at two and four weeks after transplantation. In regards to the percentage of Sox2+/DAPI+ cells, the results were also comparable for NPC and organoid transplants with ∼30% cells expressing Sox2 at both time points (Fig. 6*C*,*D*). However, as the surviving NPC grafts markedly shrunk in size from two to four weeks after grafting, the total number of Sox2+ neuroprogenitors in the NPC grafts became markedly smaller; in contrast, there was a stable and sizable NSC pool in the organoid transplants at both time points (Fig. 6*C*). Similar to the Ki67 results, the Sox2+ cells also became scattered in the grafts, unlike in stage-matched cultured cerebral organoids where Sox2+ cells resided in the VZ/SVZ aligning ventricle-like structures (Fig. 7*A*). It is also worth mentioning that the host cortex also contains numerous Sox2+ oligodendrocyte precursor cells (OPCs).

**Figure 8.**
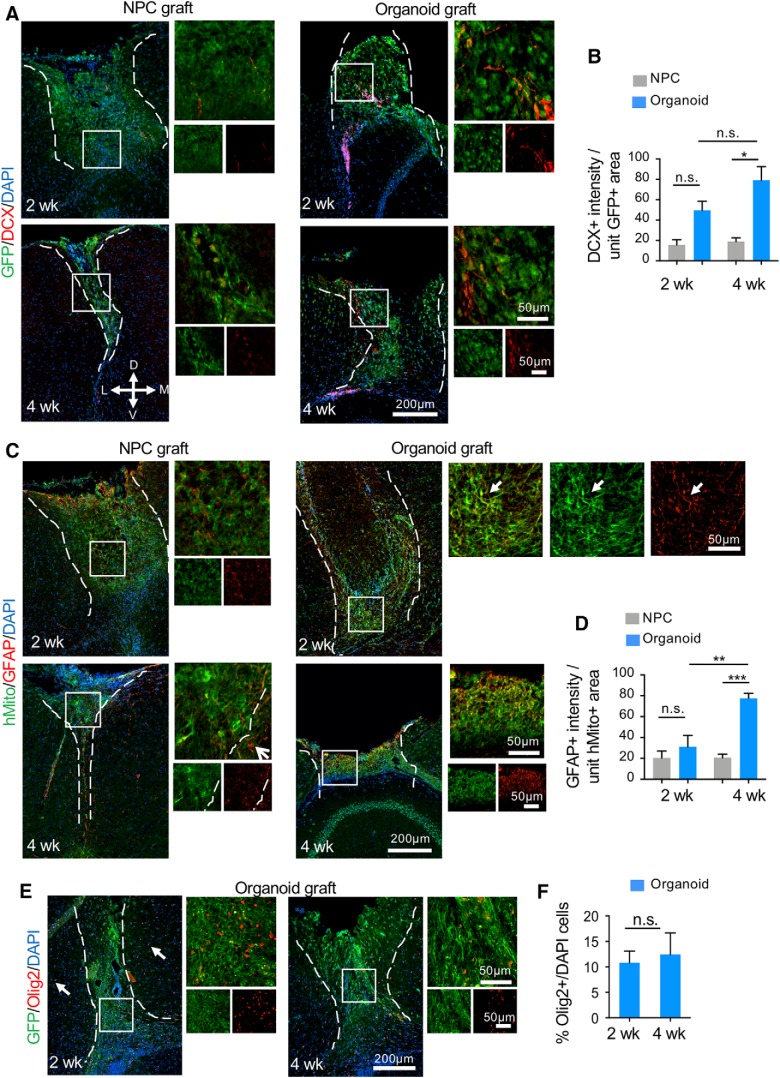
Neurodifferentiation of the engrafted C-organoids. ***A***, Representative immunofluorescent images of DCX-positive neuroblasts in NPC (left panels) and cerebral organoid transplants (right panels) at the indicated time points. D: dorsal, V: ventral, M: medial, L: lateral. ***B***, Quantification of DCX immunointensity per unit area of GFP+ grafts. A significant stronger staining intensity of DCX was measured in organoid compared to NPC transplants after four weeks. ***C***, Representative immunofluorescence images of GFAP+ cells in hMito-labeled NPC (left) and organoids grafts (right) at the indicated time points. White arrows denote colocalization of hMito and GFAP markers in transplants. ***D***, Quantification of GFAP immunointensity per unit area of hMito+ grafts. A significant stronger staining intensity of GFAP was measured in organoid transplants after four weeks. ***E***, Representative immunofluorescence images of Olig2+ cells in the engrafted cerebral organoids at the indicated time points. White arrows: Olig2+ cells in host cortical tissue. ***F***, Quantification shows no significant difference in the percentage of Olig2+/DAPI+ cells in the organoid grafts between two and four weeks after grafting. Dashed white lines delineate the graft areas. Enlarged images of the boxed area are shown on the right; *p < 0.05. **p < 0.01. ***p < 0.001; n.s., non-statistically significant. Two-way ANOVA followed by a Tukey *post hoc* test and Student’s *t* test; *n* = 3 mice for each time point and two images from each mouse.

We next compared neurodifferentiation in the two graft types. We detected significantly more DCX+ neuroblasts per unit area of GFP+ organoid transplants than NPC transplants at four weeks after transplantation (*p* = 0.03; Fig. 8*A*,*B*). This indicates enhanced survival and neuronal differentiation in the engrafted organoids. Temporal analysis showed comparable numbers of DCX+ neuroblasts within the organoid transplants at two and four weeks after grafting (*p* = 0.17). The DCX+ cells did not seem to follow any particular pattern in relationship to ventricular proximity in host brains. Astrocyte differentiation also appeared significantly more prominent in organoid transplants than in NPC grafts at four weeks after transplantation (*p* = 0.0006), although at two weeks, the expression levels of GFAP per unit graft area were comparable between the two graft types (Fig. 8*C*,*D*). Temporal comparison showed a significant increase of GFAP expression within organoid transplants between two and four weeks after grafting (*p* = 0.004). It is worth noting the central location of GFAP+ cells within the grafts and the colocalization of GFAP and hMito immunofluorescence, which support donor origin of the astrocytes (as opposed to infiltrating host reactive astrocytes). Finally, the presence of the oligodendrocyte lineage was examined by Olig2 immunofluorescence. We observed an average of 10.8% and 12.4% of cells expressing Olig2 in organoid grafts at two and four weeks after transplantation, respectively (Fig. 8*E*,*F*). In stage-matched cultured cerebral organoids, there were abundant DCX+ neuroblasts in organoids at both six and eight weeks of culture, scant GFAP+ astrocytes at eight weeks of rotary culture, but no detectable Olig2+ cells at either six or eight weeks (Fig. 7*B*).

**Figure 9.**
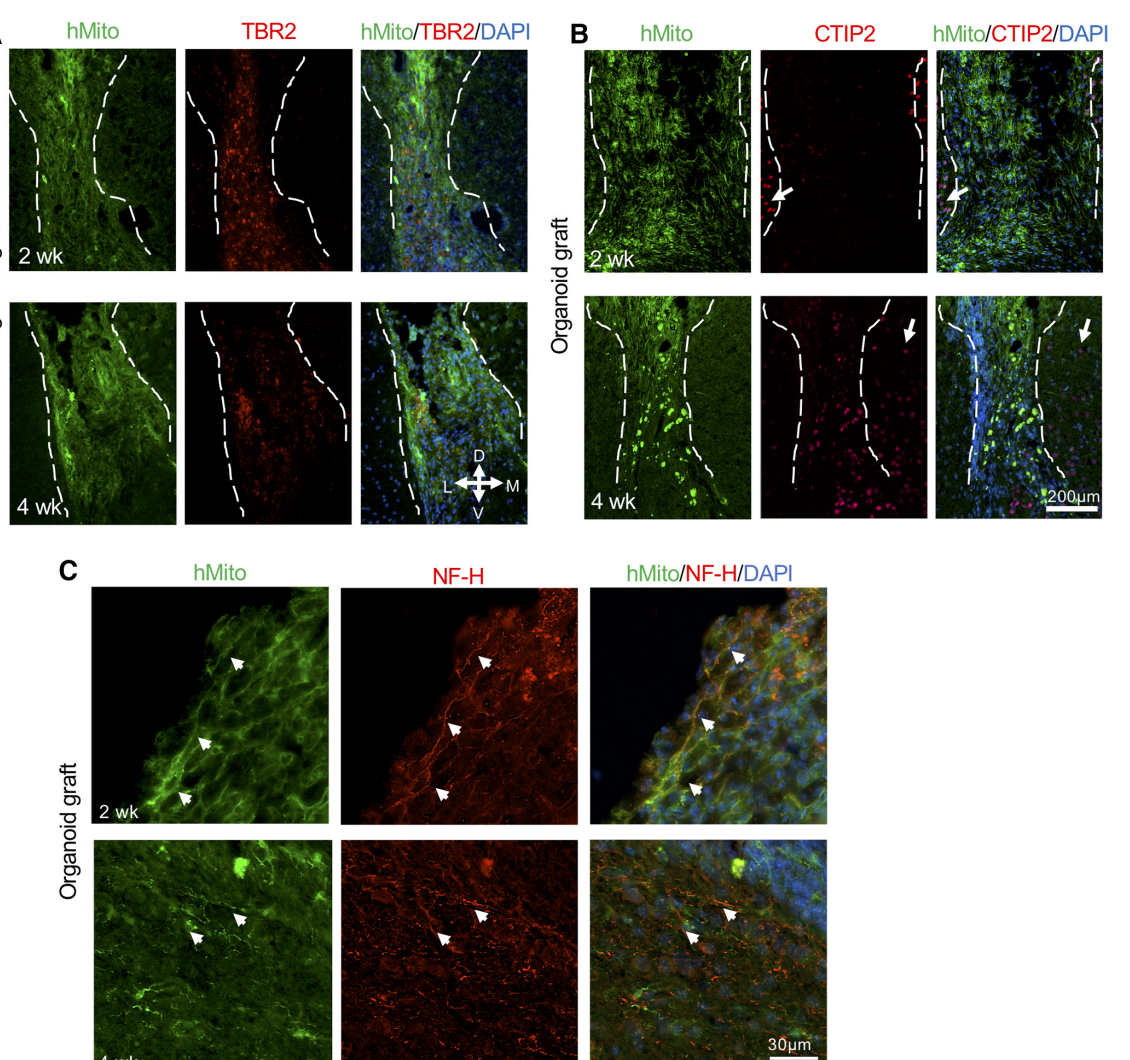
Differentiation of cerebral organoid transplants. ***A***, Representative immunofluorescence images showing cells expressing intermediate progenitor marker TBR2 in the engrafted cerebral organoids at two and four weeks after transplantation. Notice no TBR2+ cells were observed in host cortical tissue and a slight decline of the number of TBR2+ cells in the hMito+ organoid grafts from two to four weeks after transplantation. ***B***, Representative immunofluorescence images showing cells expressing deep layer neuronal marker CTIP2 in the engrafted cerebral organoids at two and four weeks after transplantation. Notice CTIP2+ cells in neighboring host cortex (white arrows), while in the hMito+ organoid grafts, there was an increase of CTIP2+ cells from two to four weeks after transplantation. ***C***, Representative immunofluorescence images show expression of NF-H in the transplanted organoids after two and four weeks. White arrowheads denote colocalization of NH-H and hMito in organoid transplants. Dashed white lines delineate the graft areas. D: dorsal, V: ventral, M: medial, L: lateral.

Further characterization of neuronal differentiation in the engrafted cerebral organoids showed the presence of cells expressing TBR2 (also known as EOMES), a marker for intermediate progenitor cells (Englund et al., 2005), which appeared more abundant at two weeks as compared to four weeks after grafting (Fig. 9*A*). Notably, there were no TBR2-expressing cells in the host cortex. We also observed cells expressing CTIP2, a marker corresponding to deep layer neurons, particularly at four weeks after grafting (Fig. 9*B*). These data are in agreement with the temporal pattern of lineage progression of neuronal differentiation; however, no layer organizations of CTIP2-positive neurons were detected in the organoid transplants, unlike in stage-matched cerebral organoid cultures (Fig. 7*A*).

To further assess the presence of mature neurons in different transplants and examine axonal growth and projections, we performed immunostaining for the neurofilament heavy chain (NF-H). While no NF-H immunosignals were detected at two or four weeks after NPC transplantation (data no shown), there was colocalization of NF-H and hMito immunosignals in organoid transplants at both time points, some with long projections (Fig. 9*C*). However, no organized projection pattern was detected, which may reflect the short time frame of the current experimental paradigm. In stage-matched cerebral organoid cultures, we also detected only low NF-H+ immunosignals (Fig. 7*B*).

## Discussion

In conventional stem cell transplantation approaches, dissociated stem cells are deposited in suspension through intracerebral, intravenous, intraarterial, or transnasal routes, all of which expose the transplanted cells to immediate hostile elements in the host brain, resulting in poor survival (Bliss et al., 2007; Chen et al., 2011). Here, we conducted side-by-side comparison of transplanting human cerebral organoids versus dissociated human NPC that were derived from the same batch of hESCs and into identical cortical locations. We demonstrated enhanced survival of organoid grafts as compared to NPC transplants, thus supporting the notion that grafting donor cells in tissue-like cerebral organoids with 3D cytoarchitecture is an essential step to enhance graft viability by providing a protective shield against hostile host elements.

It is noteworthy of the technically challenges of the current study, which entails creating a large cavity in a small host mouse brain and grafting a relatively large human cerebral organoid; in comparison, stereotactic injection of dissociated stem cells is less traumatic. The use of adult mice as recipients may ease some of the technical difficulties, and a recent study successfully demonstrated intracranial transplant of human cerebral organoids into adult mice brains (Mansour et al., 2018). However, the different study design of the two studies and the different time frame of post-transplant analyses warrant careful comparison regarding the potential influences of transplant location, the age of the host mice, SCID versus immunocompetent recipient mice on the survival and development of organoid grafts. Despite these differences, many similar findings emerge from the two studies that echo each other. First, both studies found extensive vascularization from host brain into organoid grafts by 14 d after transplantation. Second, engraftment speed and expansion of the organoid grafts appeared comparable in both studies with overall reduction in graft size from day 0 to day 14 before vascularization takes place. Third, in both studies, human organoid grafts contained Iba1+ microglia that were of host origin. Four, both studies found limited programmed cell death in the organoid grafts in contrast to the massive apoptosis seen in stage-matched organoids in culture, which may be a result of robust vascularization and *in vivo* phagocytic clearing. Fifth, the time frames of neuronal and glial differentiation in engrafted cerebral organoids were similar in both studies, with scant axonal processes and low number of astrocyte differentiation by 14 d, which then increased in abundance over time.

A good vascularization is key for survival and development of grafted tissue (Casper et al., 2003; Bates et al., 2016; Péron et al., 2017). Remarkably, two weeks after transplantation, both NPC and organoid transplants have already attracted robust penetration of host vessels into the grafts, suggesting abundant vascular growth factors released from neuroprogenitors in the grafts. It also indicates that the poor survival of NPC transplants after four weeks cannot be simply explained by lack of vascularization. One major difference between the two graft types is a much more organized cellular arrangement and tissue-like cytoarchitecture in organoid transplants, in contrast to the random organization and dissociated state of donor cells in NPC transplants. Indeed, earlier studies showed that transplantation of tissue pieces results in better survival compared to cell suspension grafts (Clarkson et al., 1998; Sekine et al., 2011). 3D, cell-cell support from multiple cell types, and cell–matrix interaction all help to improve the success of tissue transplants (Tate et al., 2009). Hence, akin to tissue-based transplantation, cerebral organoid transplants likely confer better protection and trophic support for donor cells as a result of proper cytoarchitecture. Furthermore, donor cells in the organoid transplants continue to benefit from exposure to intrinsic patterning cues that are concordant with developmental stages, while the stem cell niches in the progenitor zone of cerebral organoids can provide proper biomechanical properties and spatial cues important for donor cell survival, proliferation, and lineage progression. This is reflected by a steady fraction of proliferative cells and enhanced multilineage neurodifferentiation in the organoid transplants. In addition, as we used P8–P10 mice as recipients and relatively large sized organoid transplants, the survival of our organoid grafts may have also benefited from spatial proximity to the lateral ventricles and the retrosplenial cortex with rich vascularized surfaces (Mansour et al., 2018).

NSC therapies have been shown to improve functional outcome in a variety of CNS disease models, and the benefits have largely been attributed to trophic support or immunomodulation (Pluchino and Martino, 2008; Fainstein et al., 2013). In cerebral organoid transplants, we detected a sustained NSC pool, which by itself may significantly contribute to tissue repair. It is worth noting that the spatial organization of neuroprogenitors along ventricles in the cerebral organoids was largely lost by day 14 following transplantation, as shown by the scattering patterns of Sox2+ cells and Ki67+ cells throughout the grafts. Regardless, compared to NPC transplanted in suspension, donor neuroprogenitor cells in organoid grafts would still benefit from a microenvironment with 3D and *in vivo*-like cytoarchitecture. Regarding neurodifferentiation, there is increased progeny cells of neuronal lineage in the organoid grafts with DCX+ neuroblasts and CTIP+ cortical neurons. Astrocyte differentiation in the organoid transplants appears increased between two and four weeks after grafting, consistent with the developmental timeline of gliogenesis, which occurs after the main phase of neurogenesis (Ge et al., 2012). The number of GFAP+ cells in organoid grafts appears more abundant than in stage-matched organoid cultures (Fig. 7*B*). This may reflect stronger glial differentiation signals at the transplant site *in vivo* than in cultured organoids. Concordantly, as compared to *in vitro* cerebral organoid cultures where oligodendrocytes were detected only at later stages (Renner et al., 2017; Matsui et al., 2018), we detected ∼10% donor cells expressing Olig2 at both two and four weeks after grafting. Of note, during human neurodevelopment, Olig2 is expressed in OPC but also in a subset of neuroprogenitors (Jakovcevski and Zecevic, 2005), thus the Olig2+ cells in the organoid grafts may represent both populations.

Organoid transplantations have been examined for different target organs, including lung organoids (Dye et al., 2016), photoreceptor organoids (Santos-Ferreira et al., 2016), and liver organoids (Nie et al., 2018), all of which demonstrated good engraftment and maturation of transplanted organoids. In the current study, we limited the timeframe of our analyses to two and four weeks after transplantation, as immunocompetent P8–P10 mice were used with no immunosuppressive treatment. To investigate later time points, SCID mice or immunosuppression will be necessary. Mansour et al. (2018) recently detailed neurodevelopment in transplanted cerebral organoids up to 180 d after transplant, at which time extensive axonal growth and graft-to-host axonal projections, as well as functional synapses were observed. In both the current study and the study by Mansour et al. (2018), organoids were grown in culture for 40–50 d from hESCs (approximately four weeks in Matrigel rotary condition) with largely dorsal forebrain specification, transplanting organoids of different maturity or of different regional specification by varying culture period or including different morphogen are worthwhile in future studies. Recent advancement allowed generation of region-specific neural organoids with features of neocortex (Lee et al., 2017), telencephalon (Watanabe et al., 2005), cerebellum (Muguruma et al., 2015), neural tube (Ranga et al., 2016), hippocampus (Sakaguchi et al., 2015), and neural retina (Kuwahara et al., 2015), among others (Wei et al., 2017). These specialized neural organoids open the door for further testing of homotypic transplants wherein region-specific cerebral organoids are transplanted into the corresponding region in host brain. In this regard, an earlier study showed substantially different set of efferent axon projections from transplants of homotypic embryonic motorcortex versus heterotopic embryonic visual cortical tissue into adult motor cortex (Gaillard et al., 2007). Additional studies are needed to control stem cell proliferation, as iPSC derivatives may have oncogenic potential even after differentiation (Blum and Benvenisty, 2008; Ghosh et al., 2011). Finally, ethical questions need to be discussed concerning the use and further development of more complex cerebral organoids (Lavazza and Massimini, 2018).

In summary, our study demonstrated enhanced survival and robust vascularization of human cerebral organoid transplants in lesioned frontoparietal cortex of mouse hosts. Cerebral organoid transplantations may offer a promising novel cell replacement strategy for repair of CNS injury and neurodegenerative disorders as they provide a large set of neural cell types with both neuroprogenitors and differentiated neurons in a structured organization similar to the targeted brain area.

## Synthesis

Reviewing Editor: Orly Reiner, Weizmann Institute of Science Decisions are customarily a result of the Reviewing Editor and the peer reviewers coming together and discussing their recommendations until a consensus is reached. When revisions are invited, a fact-based synthesis statement explaining their decision and outlining what is needed to prepare a revision will be listed below. The following reviewer(s) agreed to reveal their identity: Abed Mansour, Ping Wu.

This manuscript describes an experimental model where human pluripotent stem cells are differentiated into cerebral organoids or NPCs and then transplanted into the cortex of early-born mice. The authors showed that cerebral organoids survived up to 4 weeks after grafting, express neural progenitor and differentiation markers, and importantly are infiltrated by CD31+ cells, suggesting vascularization. Importantly, they compare side-by-side transplantations of NPCs vs. organoids and showed some interesting enhanced survival and differentiation. Based on the data, they concluded that organoid graft is generally better than dissociated NPCs in terms of cell survival, more proliferating cells, and enhanced differentiation. This study, although descriptive in nature, provided further evidence to support the recent notion that tissue-like organoid may be a promising alternative for neural transplantation.

Major points:

1- 1 A similar approach was described recently (Mansour et al. 2018), and the authors fail to cite this study in the introduction and the result sections. While the described cortical location and the age of mice is different from Mansour et al., the authors fail to compare their results to this recent study which, in my opinion, appears to be an important point.

1-2 There are several open fundamental questions about the survival rate of transplanted animals and success rate of the transplantation approach. What is the efficiency of the described protocol? What are the survival rates of the grafted animals and the grafted organoids? It would be important to comment on the lifespan of the grafted animals.

2- Although the survival data of cerebral organoids looks convincing, I have some concerns about the size of the grafted organoids as compared with organoids cultured in vitro. From what I can tell in most of the images and sections, the majority of grafts looks small and not intact (compared to organoids cultured in vitro), this could be in part due to cell death or technical difficulty in dissecting these brains. It would be important to clarify this point. For example, 4 weeks organoid grafts appear very small and not intact (figure 6C).

3- 1. One of the main reasons that the authors believe contributing to the enhanced survival of organoid grafts is that the organized cellular arrangement in cerebral organoids may serve as a protective shield against hostile environment. Yet, by 2 weeks after transplantation, the earliest time point in this study, tissue-like organoids has already become disorganized; which makes one of their main hypotheses a purely speculation. Examination of brain samples at an earlier time point, e.g. 1-3 days after transplantation, would provide evidence to support the authors' hypothesis.

3-2 The analysis is restricted to 4 weeks post-transplantation. It would be interesting to explore longer time points and perhaps investigate the organoids survival and differentiation at later time points.

4-1 The higher survival rate of the organoids as compared with NPCs is impressive and convincing, however this could be due at least in part to differences in the cells number. Organoids might have higher cells numbers than the injected NPCs, therefore it would be important to count the number of cells inside the organoids before transplantation and compare to the numbers of the injected NPCs.

4-2 It is unclear how many cells are in one cerebral organoid, and there is a lack of information on how to control the size of organoid from animal to animal. For NPC study, 1x105 cells were grafted. Are the number of cells of two types of grafts compatible? Could cell number of the original graft be a factor for the survival at the later time point?

5-1 Quantitative analyses of images (Figures 3 to 6) need to be described in more details. It is stated that “one to three representative images from each animal were quantified”. However it is unclear whether the quantification was performed on the whole GFP graft area or just a region of the graft area in a 14um section. If the latter, how is a region selected? These need to be clearly explained in Methods. Basically, a strategy of tissue sampling for various staining and imaging need to be clarified. Without these information, the apparently large variation in the grafts may render the quantitative analyses inaccurate.

5-2 Figure 3: in E, The CD45 data is not convincing, and stainings and images are not clear. Also, it is not clear why CD45 was used instead of Iba1, as it would mark microglia rather than microphages.

6- It is not clear how the length if CD31+ blood vessels were measured? Also it is not clear why vasculature length between NPCs and organoid grafts appear comparable despite the fact that NPCs grafts are smaller.

7- Figure 5: The effect of lower Ki67+ cells in the grafted NPCs could be due to differences in the initial cells number and therefore it would be important to count cells in the organoids before grafting to get meaningful conclusions (see also earlier point).

8- Also, Ki67 staining should be quantified as a ratio of DAPI rather than per field. Since there can be variations in density between NPCs and organoids grafts, cells per field is not accurate. This would be true also for SOX2 counting.

9- Related to Ki67, the authors compared between Ki67 in grafted and ungrafted organoids in the main text and conclude that Ki67 in the organoids transplants appeared scattered throughout the graft, reflecting disintegration of the ventricular structure following transplantation. This observation could be perhaps due to differences on the developmental stage since the authors compare different stages and could be due to advanced differentiation rather than disorganization contrary to the authors' conclusions.

10-Figure 6 would benefit from staining with NeuN, as this nuclear maturation marker would help address and quantify the differences in differentiation potential between the two graft types.

Fig. 6A, what is the relationship between grafts and the lateral ventricle? Are there more DCX found in the graft near lateral ventricle? This may be an interesting discussion point.

11- Perhaps the biggest advantage of organoid graft is the increase in neuronal survival, and the maturation. It would be important though to compare axon growth and projections from the graft towards the host.

Minor points

1- What are the insets in Fig. 2E? it does not appear to add additional information to the figure.

2- Scale bars are confusing, labeling all the pictures could help the reader.

3- There is a typo referring to figure 4 in the text as there is no “C” in the figure.

4- The authors stated in page 12 that “our finding .....support the hypothesis that tissue-based transplanted provides better protection of donor cells from the hostile graft microenvironment” It is not clear to what data in the manuscript support that the “protection” form hostile is the reason for the enhanced survival rather than internal properties of the organoid ?

5- Methods: Stereotaxic coordinates for NPC grafting need to be confirmed for targeting the frontoparietal cortex as shown in Fig. 1A. Should “1 mm posterior to the Bregma” be “anterior to the Bregma”?

6- P.5, “exogenous pattering cues” should be “patterning”.

## References

[B1] Barker RA, Barrett J, Mason SL, Björklund A (2013) Fetal dopaminergic transplantation trials and the future of neural grafting in Parkinson’s disease. Lancet Neurol 12:84–91. 10.1016/S1474-4422(12)70295-8 23237903

[B2] Bates KA, Drummond ES, Cozens GS, Harvey AR (2016) Vascular insufficiency, not inflammation, contributes to chronic gliosis in a rat CNS transplantation model. Restor Neurol Neurosci 34:313–323. 10.3233/RNN-150591 26890100

[B3] Bliss T, Guzman R, Daadi M, Steinberg GK (2007) Cell transplantation therapy for stroke. Stroke 38:817–826. 10.1161/01.STR.0000247888.25985.62 17261746

[B4] Blum B, Benvenisty N (2008) The tumorigenicity of human embryonic stem cells. Adv Cancer Res 100:133–158. 18620095 10.1016/S0065-230X(08)00005-5

[B5] Casper D, Finkelstein E, Goldstein IM, Palencia D, Yunger Y, Pidel A (2003) Dopaminergic neurons associate with blood vessels in neural transplants. Exp Neurol 184:785–793. 10.1016/S0014-4886(03)00336-4 14769371

[B6] Chen H, Yoshioka H, Kim GS, Jung JE, Okami N, Sakata H, Maier CM, Narasimhan P, Goeders CE, Chan PH (2011) Oxidative stress in ischemic brain damage: mechanisms of cell death and potential molecular targets for neuroprotection. Antioxid Redox Signal 14:1505–1517. 10.1089/ars.2010.3576 20812869 PMC3061196

[B7] Clarkson ED, Zawada WM, Adams FS, Bell KP, Freed CR (1998) Strands of embryonic mesencephalic tissue show greater dopamine neuron survival and better behavioral improvement than cell suspensions after transplantation in parkinsonian rats. Brain Res 806:60–68. 9739108 10.1016/s0006-8993(98)00717-3

[B8] Das GD, Altman J (1972) Studies on the transplantation of developing neural tissue in the mammalian brain. I. Transplantation of cerebellar slabs into the cerebellum of neonate rats. Brain Res 38:233–49. 5028527 10.1016/0006-8993(72)90710-x

[B9] Dye BR, Dedhia PH, Miller AJ, Nagy MS, White ES, Shea LD, Spence JR (2016) A bioengineered niche promotes in vivo engraftment and maturation of pluripotent stem cell derived human lung organoids. Elife 5: pii: e19732. 10.7554/elife.19732 27677847 PMC5089859

[B10] Englund C, Fink A, Lau C, Pham D, Daza RAM, Bulfone A, Kowalczyk T, Hevner RF (2005) Pax6, Tbr2, and Tbr1 are expressed sequentially by radial glia, intermediate progenitor cells, and postmitotic neurons in developing neocortex. J Neurosci 25:247–251. 10.1523/JNEUROSCI.2899-04.2005 15634788 PMC6725189

[B11] Espuny-Camacho I, Michelsen KA, Gall D, Linaro D, Hasche A, Bonnefont J, Bali C, Orduz D, Bilheu A, Herpoel A, Lambert N, Gaspard N, Péron S, Schiffmann SN, Giugliano M, Gaillard A, Vanderhaeghen P (2013) Pyramidal neurons derived from human pluripotent stem cells integrate efficiently into mouse brain circuits in vivo. Neuron 77:440–456. 10.1016/j.neuron.2012.12.011 23395372

[B12] Fainstein N, Einstein O, Cohen ME, Brill L, Lavon I, Ben-Hur T (2013) Time limited immunomodulatory functions of transplanted neural precursor cells. Glia 61:140–149. 10.1002/glia.22420 23001547

[B14] Forsberg SL, Ilieva M, Maria Michel T (2018) Epigenetics and cerebral organoids: promising directions in autism spectrum disorders. Transl Psychiatry 8:14. 10.1038/s41398-017-0062-x 29317608 PMC5802583

[B15] Gaillard A, Prestoz L, Dumartin B, Cantereau A, Morel F, Roger M, Jaber M (2007) Reestablishment of damaged adult motor pathways by grafted embryonic cortical neurons. Nat Neurosci 10:1294–1299. 10.1038/nn1970 17828256

[B16] Ge WPP, Miyawaki A, Gage FH, Jan YN, Jan LY (2012) Local generation of glia is a major astrocyte source in postnatal cortex. Nature 484:376–380. 10.1038/nature10959 22456708 PMC3777276

[B17] Ghosh Z, Huang M, Hu S, Wilson KD, Dey D, Wu JC (2011) Dissecting the oncogenic and tumorigenic potential of differentiated human induced pluripotent stem cells and human embryonic stem cells. Cancer Res 71:5030–5039. 10.1158/0008-5472.CAN-10-4402 21646469 PMC3138859

[B18] Grade S, Götz M (2017) Neuronal replacement therapy: previous achievements and challenges ahead. NPJ Regen Med 2:29. 10.1038/s41536-017-0033-0 29302363 PMC5677983

[B19] Jakovcevski I, Zecevic N (2005) Olig transcription factors are expressed in oligodendrocyte and neuronal cells in human fetal CNS. J Neurosci 25:10064–10073. 10.1523/JNEUROSCI.2324-05.2005 16267213 PMC6725798

[B20] Johann V, Schiefer J, Sass C, Mey J, Brook G, Krüttgen A, Schlangen C, Bernreuther C, Schachner M, Dihné M, Kosinski CM (2007) Time of transplantation and cell preparation determine neural stem cell survival in a mouse model of Huntington’s disease. Exp Brain Res 177:458–470. 10.1007/s00221-006-0689-y 17013619

[B21] Kordower JH, Freeman TB, Snow BJ, Vingerhoets FJG, Mufson EJ, Sanberg PR, Hauser RA, Smith DA, Nauert GM, Perl DP, Olanow CW (1995) Neuropathological evidence of graft survival and striatal reinnervation after the transplantation of fetal mesencephalic tissue in a patient with Parkinson’s disease. N Engl J Med 332:1118–1124. 10.1056/NEJM199504273321702 7700284

[B22] Kuwahara A, Ozone C, Nakano T, Saito K, Eiraku M, Sasai Y (2015) Generation of a ciliary margin-like stem cell niche from self-organizing human retinal tissue. Nat Commun 6:6286. 10.1038/ncomms7286 25695148

[B23] Lancaster MA, Knoblich JA (2014) Generation of cerebral organoids from human pluripotent stem cells. Nat Protoc 9:2329–2340. 10.1038/nprot.2014.158 25188634 PMC4160653

[B24] Lancaster MA, Renner M, Martin CA, Wenzel D, Bicknell LS, Hurles ME, Homfray T, Penninger JM, Jackson AP, Knoblich JA (2013) Cerebral organoids model human brain development and microcephaly. Nature 501:373–379. 10.1038/nature12517 23995685 PMC3817409

[B25] Lavazza A, Massimini M (2018) Cerebral organoids: ethical issues and consciousness assessment. J Med Ethics 44:606–610. 29491041 10.1136/medethics-2017-104555

[B26] Lazic SE, Barker RA (2003) The future of cell-based transplantation therapies for neurodegenerative disorders. J Hematother Stem Cell Res 12:635–642. 10.1089/15258160360732669 14977473

[B27] Lee CT, Chen J, Kindberg AA, Bendriem RM, Spivak CE, Williams MP, Richie CT, Handreck A, Mallon BS, Lupica CR, Lin DT, Harvey BK, Mash DC, Freed WJ (2017) CYP3A5 mediates effects of cocaine on human neocorticogenesis: studies using an in vitro 3D self-organized hPSC model with a single cortex-like unit. Neuropsychopharmacology 42:774–784. 10.1038/npp.2016.156 27534267 PMC5240177

[B28] Li R, Sun L, Fang A, Li P, Wu Q, Wang X (2017) Recapitulating cortical development with organoid culture in vitro and modeling abnormal spindle-like (ASPM related primary) microcephaly disease. Protein Cell 8:823–833. 10.1007/s13238-017-0479-2 29058117 PMC5676597

[B29] Lindvall O, Kokaia Z, Martinez-Serrano A (2004) Stem cell therapy for human neurodegenerative disorders-how to make it work. Nat Med 10[Suppl.]:S42–S50. 10.1038/nm1064 15272269

[B30] Mansour AA, Gonçalves JT, Bloyd CW, Li H, Fernandes S, Quang D, Johnston S, Parylak SL, Jin X, Gage FH (2018) An in vivo model of functional and vascularized human brain organoids. Nat Biotechnol 36:432–441. 10.1038/nbt.4127 29658944 PMC6331203

[B31] Matsui TK, Matsubayashi M, Sakaguchi YM, Hayashi RK, Zheng C, Sugie K, Hasegawa M, Nakagawa T, Mori E (2018) Six-month cultured cerebral organoids from human ES cells contain matured neural cells. Neurosci Lett 670:75–82. 10.1016/j.neulet.2018.01.040 29398520

[B32] Muguruma K, Nishiyama A, Kawakami H, Hashimoto K, Sasai Y (2015) Self-organization of polarized cerebellar tissue in 3D culture of human pluripotent stem cells. Cell Rep 10:537–550. 10.1016/j.celrep.2014.12.051 25640179

[B33] Nie YZ, Zheng YW, Ogawa M, Miyagi E, Taniguchi H (2018) Human liver organoids generated with single donor-derived multiple cells rescue mice from acute liver failure. Stem Cell Res Ther 9:5. 10.1186/s13287-017-0749-1 29321049 PMC5763644

[B34] Péron S, Droguerre M, Debarbieux F, Ballout N, Benoit-Marand M, Francheteau M, Brot S, Rougon G, Jaber M, Gaillard A (2017) A delay between motor cortex lesions and neuronal transplantation enhances graft integration and improves repair and recovery. J Neurosci 37:1820–1834. 10.1523/JNEUROSCI.2936-16.2017 28087762 PMC6589972

[B35] Pluchino S, Martino G (2008) Neural stem cell-mediated immunomodulation: repairing the haemorrhagic brain. Brain 131:604–605. 10.1093/brain/awn015 18292080

[B36] Qian X, Nguyen HN, Song MM, Hadiono C, Ogden SC, Hammack C, Yao B, Hamersky GR, Jacob F, Zhong C, Yoon KJ, Jeang W, Lin L, Li Y, Thakor J, Berg DA, Zhang C, Kang E, Chickering M, Nauen D, et al. (2016) Brain-region-specific organoids using mini-bioreactors for modeling ZIKV exposure. Cell 165:1238–1254. 10.1016/j.cell.2016.04.032 27118425 PMC4900885

[B37] Ranga A, Girgin M, Meinhardt A, Eberle D, Caiazzo M, Tanaka EM, Lutolf MP (2016) Neural tube morphogenesis in synthetic 3D microenvironments. Proc Natl Acad Sci USA 113:E6831–E6839. 10.1073/pnas.1603529113 27742791 PMC5098636

[B38] Renner M, Lancaster MA, Bian S, Choi H, Ku T, Peer A, Chung K, Knoblich JA (2017) Self-organized developmental patterning and differentiation in cerebral organoids. EMBO J 36:1316–1329. 10.15252/embj.201694700 28283582 PMC5430225

[B39] Sakaguchi H, Kadoshima T, Soen M, Narii N, Ishida Y, Ohgushi M, Takahashi J, Eiraku M, Sasai Y (2015) Generation of functional hippocampal neurons from self-organizing human embryonic stem cell-derived dorsomedial telencephalic tissue. Nat Commun 6:8896. 10.1038/ncomms9896 26573335 PMC4660208

[B40] Santos-Ferreira T, Völkner M, Borsch O, Haas J, Cimalla P, Vasudevan P, Carmeliet P, Corbeil D, Michalakis S, Koch E, Karl MO, Ader M (2016) Stem cell–derived photoreceptor transplants differentially integrate into mouse models of cone-rod dystrophy. Investig Opthalmology Vis Sci 57:3509 10.1167/iovs.16-19087 27367586

[B41] Schindelin J, Arganda-Carreras I, Frise E, Kaynig V, Longair M, Pietzsch T, Preibisch S, Rueden C, Saalfeld S, Schmid B, Tinevez J-Y, White DJ, Hartenstein V, Eliceiri K, Tomancak P, Cardona A (2012) Fiji: an open-source platform for biological-image analysis. Nat Methods 9:676–682. 10.1038/nmeth.2019 22743772 PMC3855844

[B42] Sekine H, Shimizu T, Dobashi I, Matsuura K, Hagiwara N, Takahashi M, Kobayashi E, Yamato M, Okano T (2011) Cardiac cell sheet transplantation improves damaged heart function via superior cell survival in comparison with dissociated cell injection. Tissue Eng Part A 17:2973–2980. 10.1089/ten.tea.2010.0659 21875331

[B43] Snyder EY, Yoon C, Flax JD, Macklis JD (1997) Multipotent neural precursors can differentiate toward replacement of neurons undergoing targeted apoptotic degeneration in adult mouse neocortex. Proc Natl Acad Sci USA 94:11663–11668. 9326667 10.1073/pnas.94.21.11663 PMC23575

[B44] Takahashi K, Yamanaka S (2006) Induction of pluripotent stem cells from mouse embryonic and adult fibroblast cultures by defined factors. Cell 126:663–676. 10.1016/j.cell.2006.07.024 16904174

[B45] Tate CC, Shear DA, Tate MC, Archer DR, Stein DG, LaPlaca MC (2009) Laminin and fibronectin scaffolds enhance neural stem cell transplantation into the injured brain. J Tissue Eng Regen Med 3:208–217. 10.1002/term.154 19229887

[B46] Thompson LH, Björklund A (2015) Reconstruction of brain circuitry by neural transplants generated from pluripotent stem cells. Neurobiol Dis 79:28–40. 10.1016/j.nbd.2015.04.003 25913029

[B47] Watanabe K, Kamiya D, Nishiyama A, Katayama T, Nozaki S, Kawasaki H, Watanabe Y, Mizuseki K, Sasai Y (2005) Directed differentiation of telencephalic precursors from embryonic stem cells. Nat Neurosci 8:288–296. 10.1038/nn1402 15696161

[B48] Watanabe M, Buth JE, Vishlaghi N, de la Torre-Ubieta L, Taxidis J, Khakh BS, Coppola G, Pearson CA, Yamauchi K, Gong D, Dai X, Damoiseaux R, Aliyari R, Liebscher S, Schenke-Layland K, Caneda C, Huang EJ, Zhang Y, Cheng G, Geschwind DH, et al. (2017) Self-organized cerebral organoids with human-specific features predict effective drugs to combat zika virus infection. Cell Rep 21:517–532. 10.1016/j.celrep.2017.09.047 29020636 PMC5637483

[B49] Wei N, Quan Z, Tang H, Zhu J (2017) Three-dimensional organoid system transplantation technologies in future treatment of central nervous system diseases. Stem Cells Int 2017:5682354 10.1155/2017/5682354 28904534 PMC5585580

[B50] Zou J, Maeder ML, Mali P, Pruett-Miller SM, Thibodeau-Beganny S, Chou B-K, Chen G, Ye Z, Park I-H, Daley GQ, Porteus MH, Joung JK, Cheng L (2009) Gene targeting of a disease-related gene in human induced pluripotent stem and embryonic stem cells. Cell Stem Cell 5:97–110. 10.1016/j.stem.2009.05.023 19540188 PMC2720132

